# Liquiritin and Liquiritigenin Induce Melanogenesis via Enhancement of p38 and PKA Signaling Pathways

**DOI:** 10.3390/medicines6020068

**Published:** 2019-06-22

**Authors:** Takuhiro Uto, Tomoe Ohta, Akihisa Yamashita, Shunsuke Fujii, Yukihiro Shoyama

**Affiliations:** 1 Department of Pharmacognosy, Faculty of Pharmaceutical Sciences, Nagasaki International University, 2825-7 Huis Ten Bosch, Sasebo, Nagasaki 859-3298, Japan; ohta@niu.ac.jp (T.O.); taronati@i.softbank.jp (A.Y.); shoyama@niu.ac.jp (Y.S.); 2 Department of Health and Nutrition, Faculty of Health Management, Nagasaki International University, 2825-7 Huis Ten Bosch, Sasebo, Nagasaki 859-3298, Japan; fujii@niu.ac.jp

**Keywords:** licorice, liquiritigenin, liquiritin, melanin, tyrosinase, TRP-1/2, MITF, p38, PKA

## Abstract

**Background:** Liquiritin (LQ) and its aglycone, liquiritigenin (LQG), are major flavonoids in licorice root (*Glycyrrhiza* spp.). Our preliminary screening identified LQ and LQG, which promote melanin synthesis in the melanoma cells. In this study, we investigated the molecular mechanism of melanin synthesis activated by LQ and LQG. **Methods:** Murine (B16-F1) and human (HMVII) melanoma cell lines were treated with LQ or LQG. After incubation, melanin contents, intracellular tyrosinase activity, and cell viability were evaluated. Protein levels were determined using Western blotting. **Results:** LQ and LQG activated melanin synthesis and intracellular tyrosinase activity. The induction of melanin and intracellular tyrosinase activity by LQG was higher than that by LQ. LQ and LQG induced the expression of tyrosinase, tyrosinase-related protein (TRP)-1, and TRP-2. LQ and LQG also enhanced microphthalmia-associated transcription factor (MITF) expression, and cyclic AMP-responsive element-binding protein (CREB) phosphorylation. The phosphorylation of p38 and extracellular signal-regulated kinase (ERK), but not Akt, was significantly increased by LQ or LQG. Furthermore, LQ- or LQG-mediated melanin synthesis was partially blocked by p38 inhibitor (SB203580) and protein kinase A (PKA) inhibitor (H-89); however, ERK kinase (MEK) inhibitor (U0126) and phosphatidylinositol-3-kinase (PI3K) inhibitor (LY294002) had no effect. **Conclusions:** The results suggest that LQ and LQG enhance melanin synthesis by upregulating the expression of melanogenic enzymes, which were activated by p38 and PKA signaling pathways, leading to MITF expression and CREB phosphorylation.

## 1. Introduction

Melanin is synthesized in the melanosomes of melanocytes in the basal layer of the skin epidermis [1,2]. Melanin plays an important role in determining the color of human skin and hair [1,2]. Additionally, melanin has a crucial role in protecting the skin from the harmful effects of ultraviolet radiation and diverse free radicals [3,4]. In addition, hypopigmentation, which is the result of a reduction in melanin production in skin, causes pigmentary disorders such as vitiligo, albinism, and abnormal hair problems [5,6]. Therefore, inducers of melanin synthesis are used as tanning agents or in the treatment of hair depigmentation, and several of them are used for the treatment of pigmentary disorders, such as vitiligo [7].

Melanogenesis is the physiological process resulting in the synthesis of melanin. Melanogenesis is directly regulated by three enzymes: tyrosinase, tyrosinase-related protein (TRP)-1, and TRP-2 [7,8]. Among them, tyrosinase is the rate-limiting enzyme in melanogenesis and catalyzes two different reactions: the hydroxylation of tyrosine into 3,4-dihydroxyphenylalanine (DOPA), and the oxidation of DOPA into DOPA quinone. TRP-2 acts as a dopachrome tautomerase and catalyzes the rearrangement of dopachrome to form 5,6-dihydroxyindole-2-carboxylic acid (DHICA), and TRP-1 oxidizes DHICA to produce carboxylate indolequinone [7,8,9].

Multiple signaling pathways are known to control the expression of melanogenic enzymes. At the transcription level, the expression of melanogenic enzymes is regulated by microphthalmia-associated transcription factor (MITF) via binding to the M-box motif in their promoter regions [8,10,11]. In melanocyte cells, the mitogen-activated protein kinase (MAPK) family, including p38 MAPK (p38) and extracellular signal-regulated kinase (ERK), is particularly involved in regulating MITF expression [8,11,12]. In addition, protein kinase A (PKA) signaling is also known to play crucial roles in melanogenesis. PKA activation can lead to the phosphorylation of cyclic AMP (cAMP)-responsive element-binding protein (CREB) which, in turn, enhances MITF expression [8,13,14,15]. Another signaling pathway involved in melanogenesis regulation is the phosphatidylinositol-3-kinase (PI3K)/Akt signaling. The upregulation of PI3K/Akt activates melanin synthesis by MITF expression and subsequent tyrosinase, TPR-1, and TRP-2 expression [8,16,17,18]. Therefore, the regulation of these signaling pathways has become a strategic target in the control of melanin synthesis.

**Figure 1:**
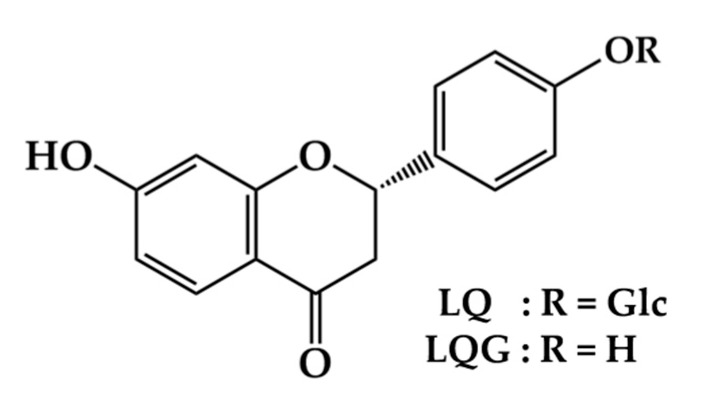
Chemical structures of liquiritin (LQ) and liquiritigenin (LQG).

Licorice root (*Glycyrrhiza* spp.) is the most important ingredient for Japanese Kampo medicine and traditional Chinese medicine, and has been reported to show various pharmaceutical functions, including anti-inflammatory, antiulcer, antiviral, antiallergenic, and liver function improvement [19,20,21,22,23,24]. Glycyrrhizin, a glycoconjugated triterpene, is one of the biologically active compounds found in licorice root, and has anti-inflammatory, antiulcer, antitumor, antiallergenic, and hepatoprotective activities [22,23,24,25,26,27,28]. Licorice root also contains a large number of flavonoid glycosides and their aglycones, which are known as biologically active components of licorice root [20,22]. Accumulated data indicate multiple biological activities of flavonoids in licorice root, such as antioxidant, antihepatotoxic, anti-inflammatory, antiulcer, antiallergenic, and antiviral activities, as well as improvement of in vitro fertilization [20,22,29,30]. Among the flavonoids in licorice root, liquiritin (LQ) and its aglycone liquiritigenin (LQG) (Figure 1) are the most important flavonoids. LQ and LQG have been demonstrated to exhibit anticancer, antidepressant, neuroprotective, myocardial cell protective, and detoxification effects, along with many other therapeutic properties [31,32,33,34,35,36,37,38]. Previously, we have prepared hybridoma cell lines that secrete monoclonal antibody against LQ and its applications for enzyme-linked immunosorbent assay (ELISA) [39]. Furthermore, an anti-LQ monoclonal antibody was generated and applied to ELISA which can be possible to determine the concentration of LQ in licorice and in various licorice-based products [40,41]. Various studies have examined the biological functions of LQ and LQG. However, there are no studies investigating the effect of LQ and LQG on melanin synthesis.

In our preliminary screening of crude drugs used in Japanese Kampo formulas for melanin synthesis property, a methanol extract of licorice root was found to exhibit melanin synthesis activity in the murine melanoma B16-F1 cell line. Among the major compounds in licorice, its major flavonoids, LQ and LQG, were found to significantly enhance melanin synthesis. In the present study, we investigated the efficacy and molecular mechanism of LQ and LQG on the regulation of melanogenesis. These findings will help elucidate the mechanism of LQ- or LQG-induced melanin synthesis.

## 2. Materials and Methods

### 2.1. Materials

LQ and LQG were purchased from Wako Pure Chemical Industries (Osaka, Japan) and Tokiwa Phytochemical (Tokyo, Japan), respectively. α-Melanocyte-stimulating hormone (α-MSH) was purchased from Sigma Chemical (St. Louis, MO, USA). Antibodies against tyrosinase, TRP-1, TRP-2, and β-actin were obtained from Santa Cruz Biotechnology (Santa Cruz, CA, USA). Antibodies against MITF, phosphorylated CREB, CREB, phosphorylated Akt, Akt, phosphorylated ERK, ERK, phosphorylated p38, and p38 were obtained from Cell Signaling Technology (Beverly, MA, USA). Fetal bovine serum (FBS) was supplied by GIBCO (Gaithersburg, MD, USA). H-89 was purchased from AdipoGen (San Diego, CA, USA). All other chemicals were obtained from Wako Pure Chemical Industries.

### 2.2. Cell Culture and Treatment

Murine melanoma B16-F1 and human melanoma HMVII cell lines were obtained from the European Collection of Authenticated Cell Cultures. B16-F1 was maintained in Dulbecco’s modified Eagle’s medium. HMVII was maintained in RPMI 1640 medium. All media were supplemented with 10% FBS and 1% penicillin–streptomycin, and then incubated at 37 °C under 5% CO_2_ in fully humidified conditions. For the cell treatment, LQ, LQG, and inhibitors were dissolved in dimethyl sulfoxide (DMSO) and stored at −20 °C before use. DMSO concentrations in the cell culture medium did not exceed 0.2% (v/v), and the controls were always treated with the same amount of DMSO as were the active compounds used in the corresponding experiments. α-MSH was dissolved in water and stored at −20 °C before use.

### 2.3. Melanin Content Assay

The cells were seeded in 24-well plates at a density of 2 × 10^4^ cells/well for B16-F1, or 5 × 10^4^ cells/well for HMVII. After incubation for 24 h, the cells were treated with LQ, LQG, or α-MSH at various concentrations for 72 h. At the end of treatment, the medium was removed, and the cells were dissolved in 120 μL of 1 M NaOH at 80 °C for 20 min. Then, absorbance was measured at 415 nm using a microplate reader (iMark, BioRad, Tokyo, Japan). Melanin content was expressed as a ratio of the control culture.

### 2.4. Cell Viability Assay

Cell viability was determined by the MTT assay as described previously [42,43,44]. In brief, B16-F1 cells were seeded in 96-well plates at a density of 0.3 × 10^4^ cells/well. After incubation for 24 h, the cells were treated with LQ or LQG at various concentrations for 72 h. At the end of treatment, 10 μL of 5 mg/mL MTT solution was added to each well, and the cells were incubated for another 4 h. The precipitated MTT formazan was dissolved with 100 μL of 0.04 N HCl–isopropanol, and the amount of formazan was measured at 595 nm using a microplate reader (iMark, BioRad, Tokyo, Japan). Cell viability was expressed as a percentage of the control culture.

### 2.5. Measurement of Mushroom Tyrosinase Activity

The effects on mushroom tyrosinase activity were determined in a cell-free system using mushroom tyrosinase [45]. Briefly, 120 μL of mushroom tyrosinase at 80.5 U/mol were used. After the addition of 20 μL sample and 70 μL L-DOPA (2.5 mM), the reaction mixture was incubated for a further 20 min at 37 °C. Tyrosinase activity was determined by the absorbance at 415 nm using a microplate reader (iMark, BioRad, Tokyo, Japan), and the mushroom tyrosinase activity was expressed as a ratio of the control value.

### 2.6. Intracellular Tyrosinase Assay

The intracellular tyrosinase activity was estimated by measuring the rate of production of dopachrome from L-DOPA [46]. B16-F1 cells were seeded in 6 cm dishes at a density of 3 × 10^5^ cells/dish. After incubation for 24 h, the cells were treated with LQ or LQG at various concentrations for 72 h. At the end of treatment, the cells were washed with ice-cold PBS twice, 600 μL of 0.1 M sodium phosphate buffer (pH 6.8) containing 1% Triton X-100, and proteinase inhibitor cocktail. The lysate was clarified by centrifugation at 12,000*g* for 15 min at 4 °C, and the supernatants were collected. A reaction mixture containing 90 μL cell lysate and 10 μL of 5 mM L-DOPA was placed into a 96-well plate. After 30–60 min incubation (according to the content of dopachrome formation) at 37 °C in the dark, the absorbance was measured at 415 nm using a microplate reader (iMark, BioRad, Tokyo, Japan). The tyrosinase activity was expressed as a ratio of the control culture by normalization based on protein concentrations.

### 2.7. Western Blotting

The cells were seeded in 6 cm dishes at a density of 3 × 10^5^ cells/dish. After incubation for 24 h, the cells were treated with LQ, LQG, or α-MSH at various concentrations. After treatment for various periods, the harvested cells were lysed, and the supernatants were boiled for 5 min. Protein concentration was determined using a dye-binding protein assay kit according to the manufacturer’s manual (Biorad, Richmond, CA, USA). Equal amounts of lysate protein were subjected to SDS-PAGE. Proteins were electrotransferred to PVDF membranes and detected as described previously [42,43,44]. The relative intensity of the indicated band was quantified using ImageJ software (1.50i), National Institutes of Health, Bethesda, MD, USA), and the value was normalized to a corresponding loading control and expressed as the fold change in the control group.

### 2.8. Statistical Analysis

The data were analyzed by ANOVA followed by Dunnett’s test using GraphPad Prism 6 software (San Diego, CA, USA). All data are presented as mean ± standard error of the mean (SEM). Each experiment was repeated at least three times and *p* < 0.05 was considered statistically significant.

## 3. Results and Discussion

### 3.1. Effects of LQ and LQG on Melanin Synthesis and Cell Viability

**Figure 2:**
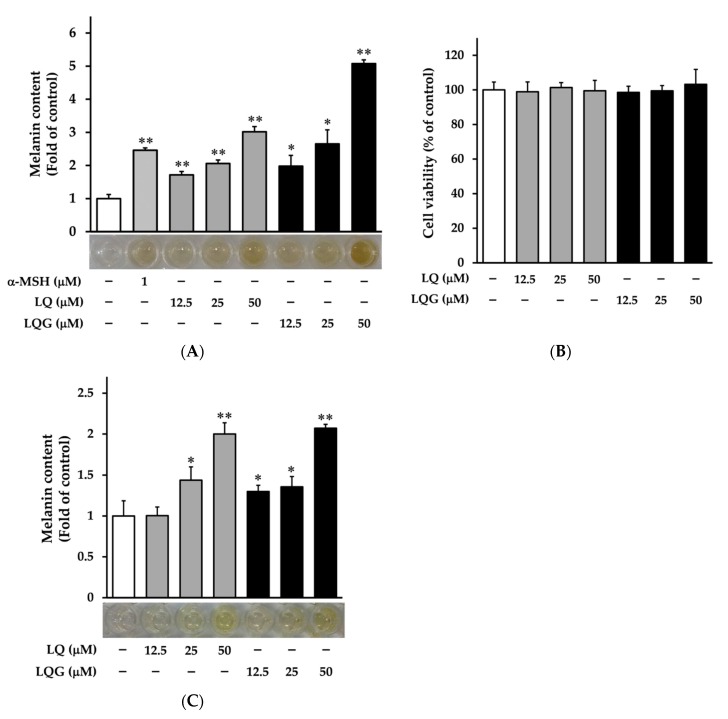
Effects of LQ and LQG on melanin synthesis and cell viability. (**A**) B16-F1 cells were treated with LQ, LQG, or α-melanocyte-stimulating hormone (α-MSH) at the indicated concentrations for 72 h, and the melanin content was determined as described in Materials and Methods. (**B**) B16-F1 cells were treated with LQ or LQG at the indicated concentrations for 72 h, and the cell viability was determined as described in Materials and Methods. There were no significant differences between the control and experimental groups (*p* > 0.05). (**C**) HMVII cells were treated with LQ or LQG at the indicated concentrations for 72 h, and the melanin content was determined as described in Materials and Methods. Values are the mean ± SEM of three independent experiments. *p < 0.05 and **p < 0.01 versus control.

First, we examined the effects of LQ and LQG on melanin synthesis in murine melanoma B16-F1 cells. The cells were treated with LQ or LQG at several doses (12.5, 25, or 50 μM) for 72 h, then the melanin content was determined using a melanin-content assay. The α-MSH was used as a positive control [47,48]. LQ and LQG significantly increased melanin content in a dose-dependent manner, as shown in Figure 2A. The color of cell pellets lysed with 1 M NaOH was darker after treatment with LQ and LQG. In addition, we measured the cytotoxicity of LQ and LQG in B16-F1 cells using the MTT assay. As shown in Figure 2B, LQ and LQG did not affect cell viability at concentrations that increased melanin content, clearly indicating that their effects were not attributable to cell proliferative activities. Furthermore, we observed the effects of LQ and LQG on melanin synthesis in human melanoma HMVII cells (Figure 2C). LQ and LQG increased melanin production and darkened the cell pellets in a similar manner as in the B16-F1 cells. Melanin synthesis by LQG was higher than that by LQ in both cell lines.

### 3.2. Effects of LQ and LQG on Tyrosinase Activity

**Figure 3:**
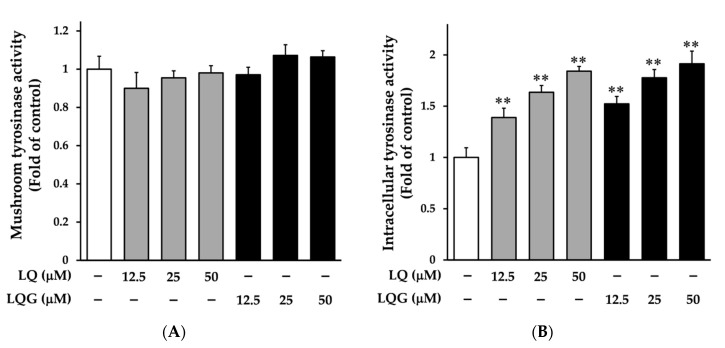
Effects of LQ and LQG on tyrosinase activity. (**A**) The effects of LQ and LQG on mushroom tyrosinase activity were determined as described in Materials and Methods. There were no significant differences between the control and experimental groups (*p* > 0.05). (**B**) B16-F1 cells were treated with LQ or LQG at the indicated concentrations for 72 h, and the intracellular tyrosinase activity was determined as described in Materials and Methods. Values are the mean ± SEM of three independent experiments. **p < 0.01 versus control.

Next, we determined the effects of LQ and LQG on tyrosinase activity using cell-free mushroom tyrosinase and cellular systems. As shown in Figure 3A, LQ and LQG had no effect on the mushroom tyrosinase activity, suggesting that the effects of LQ and LQG on melanin synthesis observed in B16-F1 and HMVII cells are not due to the direct action on the catalytic activity of tyrosinase. By contrast, LQ and LQG significantly induced the intracellular tyrosinase activity in a dose-dependent manner (Figure 3B). These results imply the possibility that LQ and LQG regulate the expression of melanogenic enzymes.

### 3.3. Effects of LQ and LQG on the Expression of Melanogenic Enzymes and MITF

**Figure 4:**
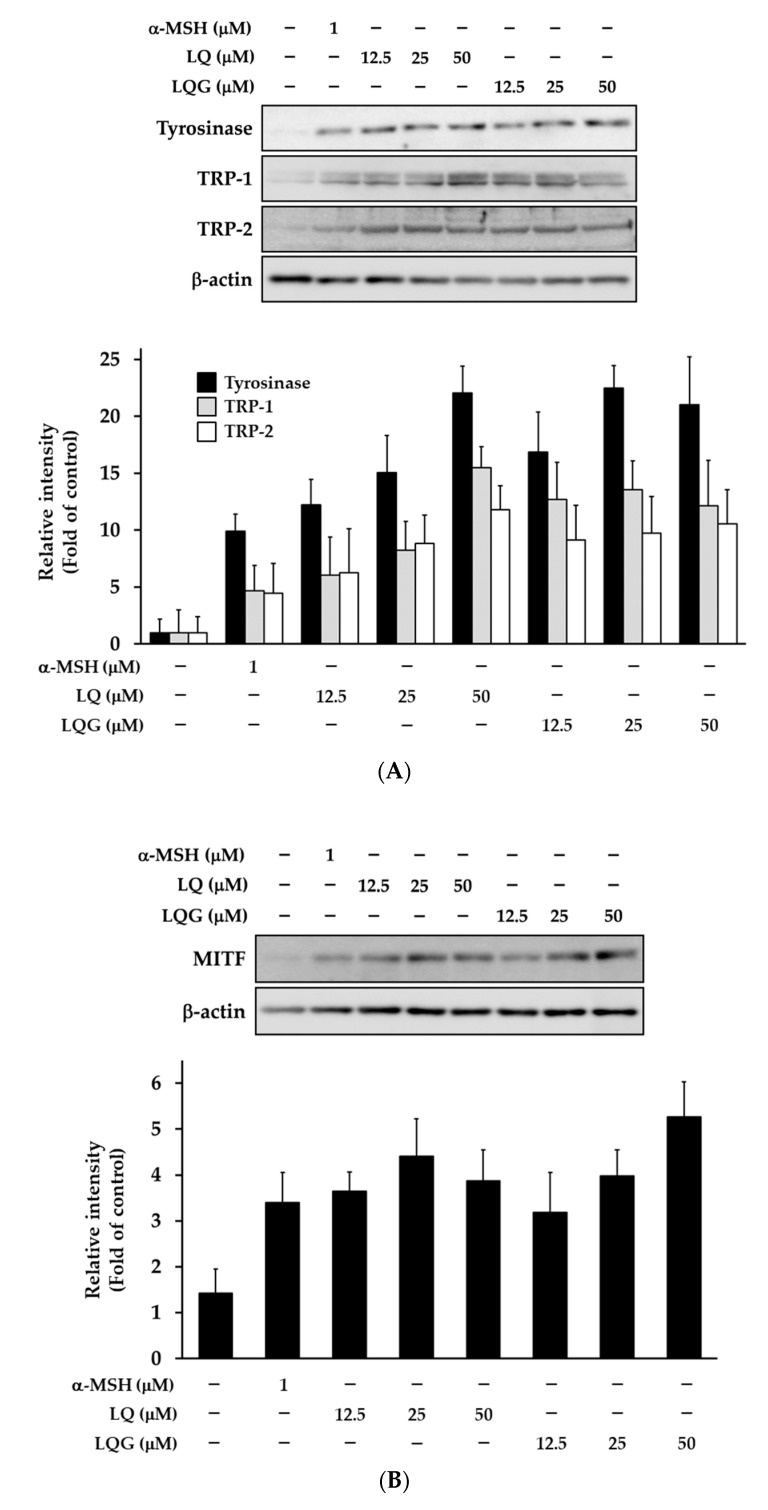
Effects of LQ and LQG on the protein levels of melanogenic enzymes (**A**) and MITF (**B**). B16-F1 cells were treated with LQ, LQG, or α-MSH at the indicated concentrations for 48 h, and the protein expressions of melanogenesis-related proteins and MITF were determined using Western blotting as described in Materials and Methods. The data shown are representative of 4–6 independent experiments.

The effects of LQ and LQG on melanogenic enzymes were determined by Western blotting. LQ and LQG clearly induced the protein levels of tyrosinase, TRP-1, and TRP-2 in a dose-dependent manner (Figure 4A). As a key transcription factor for these proteins is MITF, we examined MITF expression. As expected, the MITF level was also enhanced by LQ and LQG (Figure 4B). These results suggested that LQ- and LQG-induced melanin synthesis through the upregulation of the melanogenic enzymes and MITF at protein levels.

### 3.4. Effects of LQ and LQG on the Phosphorylation of CREB, p38, ERK, and Akt

**Figure 5:**
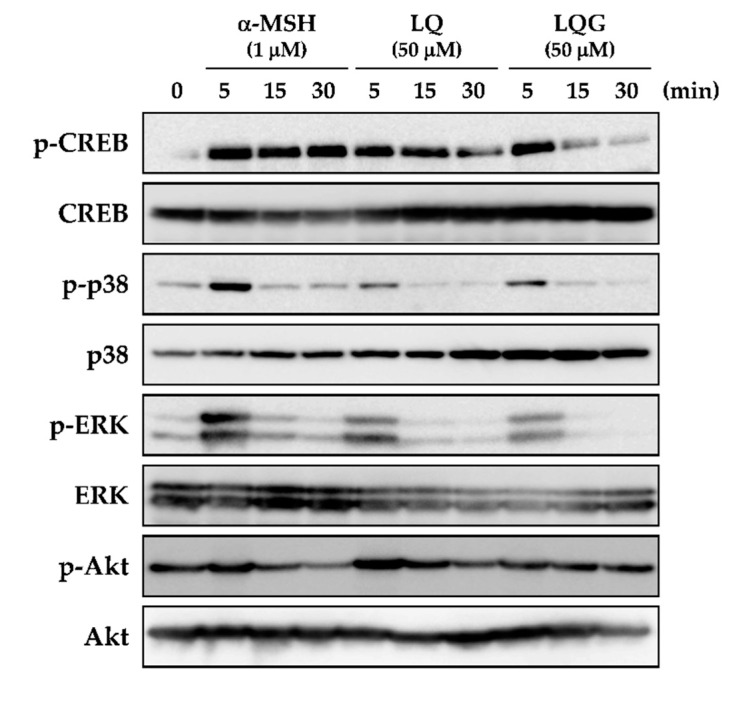
Effects of LQ and LQG on the phosphorylation of CREB, p38, ERK, and Akt. B16-F1 cells were treated with LQ, LQG, or α-MSH at the indicated concentrations for 5, 15, or 30 min, and then the protein expression levels were determined by Western blotting as described in Materials and Methods. The data shown are representative of 4–6 independent experiments.

To clarify the mechanism of LQ and LQG in melanin synthesis, we next examined CREB phosphorylation, which is involved in the protein expression of melanogenic enzymes through the upregulation of MITF expression. As shown in Figure 5, phosphorylation of CREB was not detected before treatment (0 min). However, CREB was significantly phosphorylated after 5 min treatment of LQ or LQG, as well as α-MSH.

MITF expression and CREB phosphorylation are activated by p38 and ERK, which belong to the MAPK signaling pathway [8,11,12]. Before treatment, p38 and ERK were not phosphorylated, but LQ and LQG induced the phosphorylation of p38 and ERK after 5 min treatment in the same fashion as α-MSH. Previous studies have reported that MITF was negatively regulated by the PI3K/Akt signaling pathway [49,50,51,52]. The results indicated that α-MSH clearly reduced Akt phosphorylation at 30 min, but the effects of LQ and LQG were weak compared to those of α-MSH.

### 3.5. Effects of Signaling Inhibitors on LQ- or LQG-Induced Melanin Synthesis

**Figure 6:**
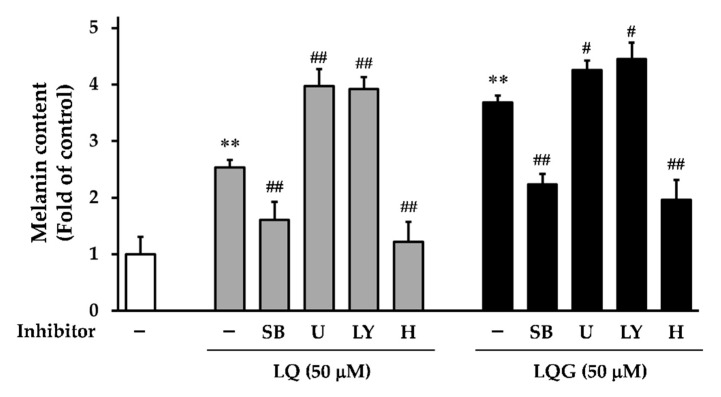
Effects of specific inhibitors on LQ- or LQG-induced melanin synthesis. B16-F1 cells were pretreated with specific inhibitors for 1 h and then treated with LQ (50 μM) or LQG (50 μM) for 72 h, and the melanin content was determined as described in Materials and Methods. Values are the mean ± SEM of three independent experiments. **p < 0.01 versus control. #*p* < 0.05 and ##*p* < 0.01 versus LQ- or LQG-treated group. LY, LY294002 (10 μM); SB, SB203580 (10 μM); U, U0126 (5 μM); H, H-89 (2.5 μM).

To further confirm the role of MAPK and PI3K/Akt pathways on LQ- or LQG-induced melanin synthesis, we used specific inhibitors, including p38 inhibitor (SB203580), ERK kinase (MEK) inhibitor (U0126), and PI3K inhibitor (LY294002). Cells were pretreated with specific inhibitors 1 h before the addition of LQ or LQG, and then incubated for 72 h for the measurement of melanin content. As shown in Figure 6, SB203580 abolished the induction of melanin synthesis by LQ and LQG, suggesting that LQ and LQG may trigger the melanin synthesis via p38 phosphorylation. By contrast, U0126 and LY294002 increased activation, rather than suppression, of melanin synthesis. These results imply that ERK phosphorylation induced by LQ and LQG was not associated with melanin synthesis, and the PI3K/Akt pathway was not involved in LQ- or LQG-induced melanin synthesis.

It is well known that the PKA signaling pathway is also involved in melanin synthesis [8,13,14,15]. PKA can be activated by the elevation of cellular cAMP, and its activation increases MITF transcriptional activity through CREB phosphorylation, resulting in the protein expression of tyrosinase, TRP-1, and TRP-2 [13,14,15]. Therefore, to determine whether the effects of LQ or LQG on melanin synthesis were also mediated by the PKA signaling pathway, we used a PKA inhibitor (H-89). Melanin synthesis induced by both LQ and LQG was strongly inhibited by H-89, suggesting that the PKA signaling pathway is also involved in LQ- or LQG-induced melanin synthesis. Taken together, these results clearly indicate that the p38 and PKA signaling pathways are necessary to evoke melanin synthesis by LQ and LQG.

## 4. Conclusions

In this study, we demonstrated that LQ and LQG have the property of melanin induction in murine and human melanoma cell lines. Our observations indicated that LQ and LQG did not directly affect the catalytic activity of tyrosinase, but upregulated the expression of melanogenic enzymes such as tyrosinase, TRP-1, and TRP-2. The upregulation of their expression might have been caused by the activation of MITF expression, which binds to the M-box motif in their promoter regions [8,10,11]. Furthermore, we identified the molecular mechanism underlying the activation of MITF expression leading to melanin induction. Both p38 and ERK were phosphorylated by LQ and LQG. Whereas the p38 inhibitor abolished LQ- or LQG-mediated melanin synthesis, the MEK inhibitor could not inhibit them. It is reported that the activation of p38 upregulates CREB phosphorylation and MITF expression, leading to melanogenesis [8,46,53,54,55]. Thus, p38 signaling may contribute to LQ- or LQG-mediated melanin synthesis induction. Similarly, we confirmed the involvement of the PI3K/Akt pathway, indicating that LQ and LQG were not involved in the PI3K/Akt pathway. The activation of PKA signaling is known to induce CREB phosphorylation and stimulate MITF transcription [8,13,14,15]. Consistent with these findings, we observed that the inhibition of PKA signaling blocked LQ- or LQG-mediated melanin synthesis, implying that PKA signaling is also involved in LQ- or LQG-induced melanin synthesis. Taken together, these results suggest that LQ and LQG stimulate both p38 and PKA signaling pathways, leading to MITF expression and CREB phosphorylation. The concentration of LQ and LQG used in this study might be relatively high, but we suppose that there is a possibility that licorice flavonoids having a similar structure of LQ or LQG can coordinately enhance melanin synthesis. Our results suggest that LQ and LQG can be used as potentially potent and safe therapeutic agents for the treatment of pigmentary disorders and tanning agents.
